# Upregulated Expression of Indoleamine 2, 3-Dioxygenase in Primary Breast Cancer Correlates with Increase of Infiltrated Regulatory T Cells *In Situ* and Lymph Node Metastasis

**DOI:** 10.1155/2011/469135

**Published:** 2011-10-24

**Authors:** Jinpu Yu, Jingyan Sun, Shizhen Emily Wang, Hui Li, Shui Cao, Yizi Cong, Juntian Liu, Xiubao Ren

**Affiliations:** ^1^Department of Immunology, Key Laboratory of Cancer Prevention and Therapy, Tianjin Cancer Institute & Hospital, Tianjin Medical University, Tianjin 300060, China; ^2^Department of Breast Cancer, Key Laboratory of Cancer Prevention and Therapy, Tianjin Cancer Institute & Hospital, Tianjin Medical University, Tianjin 300060, China; ^3^Division of Tumor Cell Biology, Beckman Research Institute of City of Hope, Duarte, CA 91010, USA

## Abstract

IDO has been reported to induce immunotolerance and promote metastasis in solid malignancy, but the mechanisms involved were not fully understood. In this study, the expression of IDO in primary breast cancer was examined and the correlation between the expression levels of IDO and the densities of Foxp3^+^ Tregs *in situ* was studied. The IDO stably-expressing CHO cells(IDO/CHO) were generated to evaluate the induction of Foxp3^+^ Tregs after coculturing with CD3^+^ T cells *in vitro*. The IDO expression in cancer was higher than that in benign diseases both at RNA and protein levels. The IDO expression was significantly upregulated in tumors of more advanced stages and with more extensive lymph node metastasis, and displayed positive linear correlation with the density of Foxp3^+^ Tregs. We further demonstrated that CD4^+^CD25^+^CD127^−^ Tregs could be amplified by coculturing CD3^+^ T cells with IDO/CHO cells *in vitro* which displayed increasing Foxp3 expression both at mRNA and protein levels. Our results implied that up-regulation of IDO in primary breast cancer may inhibit local immune surveillance and promote metastasis by favoring development and infiltration of Foxp3^+^ Tregs in the tumor microenvironment.

## 1. Introduction

Breast cancer is the most common solid malignancy in women worldwide. A substantial fraction of breast cancer patients develop distant metastases shortly after diagnosis. Metastatic breast cancer is associated with poor prognosis with shorter survival time and refractoriness to therapies. Previous studies have proposed the mechanisms of early metastasis, including overexpression of growth factor receptors and resistant to apoptosis [1, 2], downregulation of adherent molecules during epithelial-mesenchymal transition (EMT) [3–5], degradation of extracellular matrix after activation of matrix metalloproteinases (MMPs) [6, 7], enhanced tumor angiogenesis [8, 9], and inhibition of effective antitumor immunity [10, 11]. Breast cancer cells can evade the immune attack through a variety of complex mechanisms, among which tumor-derived immunosuppression resulting from upregulation of metabolistic enzymes, such as indoleamine 2,3-dioxygenase (IDO), has shown a crucial role in the recent studies [12–15]. 

IDO is a rate-limiting enzyme in the catabolic process of extrahepatic tryptophan which is an essential amino acid for T-cell proliferation and activation. Deprivation of tryptophan in the microenvironment directly affects the cytotoxicity and cytokine secretion of T cells. In addition, the toxic metabolites generated from tryptophan via the Kynurenine pathway directly induce T-cell apoptosis* in vitro *[16]. It is also reported that IDO may inhibit T-cell immunity by inducing differentiation and maturation of CD4^+^CD25^+^ regulatory T cells (Tregs) [17]. Therefore, IDO has been implicated in the development of autoimmune diseases, regulation of transplantation immunity, and maintenance of maternal-fetal tolerance [18]. 

Recent studies demonstrated that the expression level of IDO increased in many types of human tumors, including cancers of the lungs, prostate, pancreas, and cervical carcinoma. Tumor-derived IDO dramatically inhibits local T-cell-dependent antitumor immunity and facilitates tumor metastasis [19, 20]. Our previous studies, along with work from other groups, demonstrated that the proportion of CD4^+^CD25^+^ Treg subset increased in breast cancer patients, with strong correlations with the histological grade and the tumor size [21, 22]. However, it is not clear if the increase of CD4^+^CD25^+^ Tregs* in situ* is correlated with the upregulated expression of IDO in tumor cells. In this study, the expression of IDO at both mRNA and protein levels were examined in 26 cases of primary breast cancer and 10 cases of benign breast diseases. The correlation between IDO expression levels and the densities of Foxp3^+^ Tregs in the primary tumor tissues (PTs) and tumor-draining lymph nodes (TDLNs), as well as various clinical and pathological indexes of the patients were investigated. Our data indicated that the expression of IDO in breast cancer PTs was higher than that in benign disease tissue, but lower than that in TDLNs. IDO was predominantly expressed in cancer cells and modestly expressed in hyperplastic ductal cells and some myeloid cell-like karyocytes in TDLNs. The expression of IDO in PTs was positively linearly correlated to the density of Foxp3^+^ Tregs in PTs and TDLNs and was significantly higher in tumors of more advanced stages and with more extensive lymph node metastasis. In order to find out if high level of IDO can induce amplification of Foxp3^+^ Tregs, we cocultured CD3^+^ T cells with IDO^+^ CHO cells (IDO/CHO) *in vitro*. The proportion and absolute number of CD4^+^CD25^+^CD127^−^ Tregs increased after coculturing CD3^+^ T cells with IDO/CHO for 7 days, along with elevated Foxp3 expression at mRNA and protein levels in the CD3^+^ T cells. These results suggested that upregulation of IDO in breast cancer cells may lead to increased recruitment of CD4^+^CD25^+^ Tregs into the tumor microenvironment and thus inhibit the local immune surveillance and promote metastasis. Therefore, novel IDO-targeted therapies may provide a new direction for the treatment of breast cancer.

## 2. Materials and Methods

### 2.1. Patients

Fresh and paraffin-embedded samples, including primary tumors, TDLNs, and normal adjacent tissues were collected from 26 cases of breast cancer patients who were treated with radical mastectomy for breast cancer at the Department of Breast Oncology of Tianjin Cancer Institute & Hospital from June to December, 2009. All patients included 25 females and 1 male with a median age of 50 (31~70) years old, among whom 21 cases of invasive ductal carcinoma, 2 cases of invasive lobular carcinoma, 1 case of invasive micropapillary carcinoma, 1 case of mucinous carcinoma, and 1 case of secretory carcinomas were diagnosed pathologically based on the 2003 WHO classification of breast tumor. According to the 6th edition of the AJCC Cancer Staging Manual, all patients included 2 cases of stage I, 13 cases of stage IIA, 7 case of stage IIB, 3 cases of stage IIIA, and 1 case of stage IIIC. Other 10 patients with benign breast diseases, including 7 cases of breast fibroadenoma and 3 cases of lobular hyperplasia were enrolled as control. This research project was approved by the Ethics Committee of Tianjin Cancer Institute and Hospital. Written consents were obtained from each patient.

### 2.2. Immunohistochemistry (IHC)

Formaldehyde-fixed, paraffin-embedded PTs and TDLNs samples were sectioned into 4 *μ*m slices and affixed on glass slides. The immunohistochemical staining was performed according to the instruction manuals. Briefly, after being heated for half an hour at 56°C, the samples were deparaffinized in xylene and rehydrated through graded alcohol. Antigens were retrieved by heating in citrate buffer for a total of 20 minutes. Endogenous peroxidase activity was quenched in a bath of methanol and hydrogen peroxide for 30 minutes. All samples were incubated overnight at 4°C with mouse anti-human Foxp3 monoclonal antibody (Clone PCH101, eBioscience, San Diego, Calif, USA) and mouse anti-human IDO monoclonal antibody (Clone 10.1, Chemicon, Temecula, Calif, USA) at concentrations of 1 : 1000 and 1 : 500, respectively. These antibodies were detected by a biotinylated secondary antibody (goat anti-mouse IgG-HRP, sc-2302, Santa Cruz, Calif, USA) labeled with streptavidin-horseradish peroxidase (HRP), with the use of a DAB staining kit (Maixin Biotechnology, Fuzhou, China). For negative control, the primary antibody was substituted with PBS. Positive cells were stained brownish yellow in the cytoplasm (IDO-positive staining) or nucleus (Foxp3-positive staining). Two indicators were used to describe the protein expression of IDO and Foxp genes: staining rate (SR) and staining index (SI). The SRs referred to the percentages of positive samples in all samples. The SIs referred to the percentages of positively stained cells in each sample which were calculated using the following formula: (SI = number of positively stained cells/total number of counted cells ×100%). The SI was determined upon the average of at least five high-powered fields (400x magnification). An Olympus BX51 microscope was used for image acquisition and data analysis.

### 2.3. Establishment of Stable IDO^+^ CHO Transfectants

A 1225 kb fragment encoding the entire open reading frame (ORF) of human IDO gene was amplified by RT-PCR method using total RNA isolated from MDA-MB-435s breast cancer cells as template. The PCR product was firstly cloned into the pMD19-T Simple Vector (Takara, Japan) and then subcloned into the pIRES2-EGFP vector (Clontech, MountainView, Calif, USA) to generate a recombinant expression plasmid pIRES2-EGFP-IDO. The CHO cells were transfected with pIRES2-EGFP-IDO using a standard electroporation method (field strength of 350 V/cm, 60 *μ*s, 1 pulse), and IDO^+^ CHO transfectants (CHO/IDO) were selected by G418 (1 mg/mL, Invitrogen, Carlsbad, Calif, USA) in RPMI 1640 medium supplemented by 10% FBS (Hyclone, Calif, USA) as described previously [23]. CHO cells transfected with pIRES2-EGFP (CHO/EGFP) were used as negative control.

### 2.4. Coculture of CHO/IDO Cells and CD3^+^ T Cells

The CD3^+^ T cells in peripheral blood mononuclear cells (PBMCs) of breast cancer patients were purified using Human Pan T-cell Isolation Kit II (Miltenyi Biotec, Germany) according to the manufacturer's instructions. 1 × 10^5^ CHO/IDO cells and CHO/EGFP cells were seeded in a 24-well plate and cocultured with 2 × 10^6^ purified T cells in complete RPMI 1640 medium supplemented with 10% FBS and 50 U/mL rhIL-2 (PeproTech, USA) at 37°C in a 5% CO_2_ incubator. Unstimulated T cells cocultured in complete RPMI 1640 medium supplemented with 10% FBS and 50 U/mL rhIL-2 were used as control. The nonadherent T cells under different treatments were harvested 7 days later for flow cytometry analysis, quantitativee real time RT-PCR, and Western Blot analysis.

### 2.5. Flow Cytometry Analysis

The proportions and absolute counts of Tregs in T cells cocultured with CHO/IDO or CHO/EGFP cells for 7 days, as well as the control T cells were detected by flow cytometry using FITC labeled anti-human CD25, PE labeled anti-human CD127, and PerCP-Cy5.5 labeled anti-human CD4 (BD Biosciences Pharmingen, San Diego, Calif, USA) in TrueCount tubes (BD Pharmingen, San Diego, Calif). The isotype-matched IgG1 was used as negative control to eliminate nonspecific staining. 1 × 10^5^ cells were incubated with antibodies for 30 min on ice in dark. Then, the cells were washed twice with PBS containing 0.2% BSA, fixed using 1% paraformaldehyde and analyzed using a FACSAria flow cytometry (Becton Dickinson, Mountain View, Calif). At least 50,000 events were acquired for each analysis. All samples were measured at least three times.

### 2.6. Quantitative Real-Time RT-PCR (qRT-PCR) Assay

The mRNA expression of IDO gene in PTs, TDLNs, and normal adjacent tissues, as well as the mRNA expression of forehead transcription factor 3 (Foxp3) gene in stimulated and unstimulated T cells was analyzed using quantitative real-time RT-PCR. The total RNA was extracted using Trizol Reagent (Invitrogen, Carlsbad, Calif, USA) and reverse transcribed to cDNA using MMLV reverse transcriptase (Promega, Madison, Wis, USA). The expression levels of target genes were quantified using the SYBR Premix Ex Taq system (Takara Bio, Tokyo, Japan) following the manufacturer's instructions. The primers of IDO, Foxp3, and *β*-actin were listed in Table 1. The thermal cycling program was listed below: initial denaturalization at 94°C for 5 minutes, 94°C for 30 seconds, 58°C for 30 seconds, and 72°C for 45 seconds for 35 cycles; after the last cycle, 72°C for 10 minutes. The products of PCR reactions were analyzed by agarose gel electrophoresis. The relative amounts of IDO and Foxp3 genes were normalized by *β*-actin and calculated using the formula: 2^−ΔCt^(ΔCt = Ct_Foxp3_ − Ct_*β*-actin_). All tests were repeated at least four times.

### 2.7. Western Blot

The protein expression of Foxp3 in T cells cocultured with CHO/IDO or CHO/EGFP cells for 7 days, as well as in the control unstimulated T cells, was analyzed using Western Blot analysis. T cells were washed using PBS and lysed in lysis buffer (50 mM Tris-HCl, pH 7.4; 1% NP-40; 0.25% sodium deoxycholate; 150 mM NaCl, 1 mM EDTA, 1 mM Na_3_VO_4_, 1 mM PMSF, 1 mM NaF and 1 *μ*g/mL of aprotinin and leupeptin, pepstatin) on ice. After centrifugation, soluble cellular protein concentration was determined using Micro BCA Protein Assay Kit (Pierce Biotechnology, Ill, USA). The proteins were separated on SDS-PAGE and transferred to PVDF membranes. The membrane was incubated with rabbit polyclonal anti-Foxp3 antibody (BioLegend, San Diego, Calif, USA) overnight at 4°C. Then, the membrane was incubated with HRP-conjugated mouse secondary antibodies (Zhongshanjinqiao, Beijing, China) for 1 h at room temperature. Bound HRP was detected by using SuperSignal West Pico Chemiluminescent Substrate (Pierce Biotechnology, Ill, USA). The intensity of bands was recorded using the ChemiDoc XRS imaging system and analyzed using Quantity One software (Bio-Rad Laboratories, Hercules, Calif, USA).

### 2.8. Statistical Analysis

All data were presented as mean ± standard deviation (SD). The statistical analysis was performed using a SPSS 13.0 software package. The one-way single factor analysis of variance (ANOVA) was used for the comparison of the quantitative data, and the chi-square (*χ* ^2^) test was used for the comparison of the qualitative data. The Spearman's rank-order test and linear regression analysis were used to assess correlations between IDO^+^ and Foxp3^+^ SIs. The survival times were compared using Kaplan-Meier Survival analysis. The level of statistical significance was set at *P* < 0.05.

## 3. Results

### 3.1. The Expression of IDO in Breast Cancer PTs and TDLNs Was Higher Than That in Benign Diseases at Both RNA and Protein Levels

**Figure 1:**
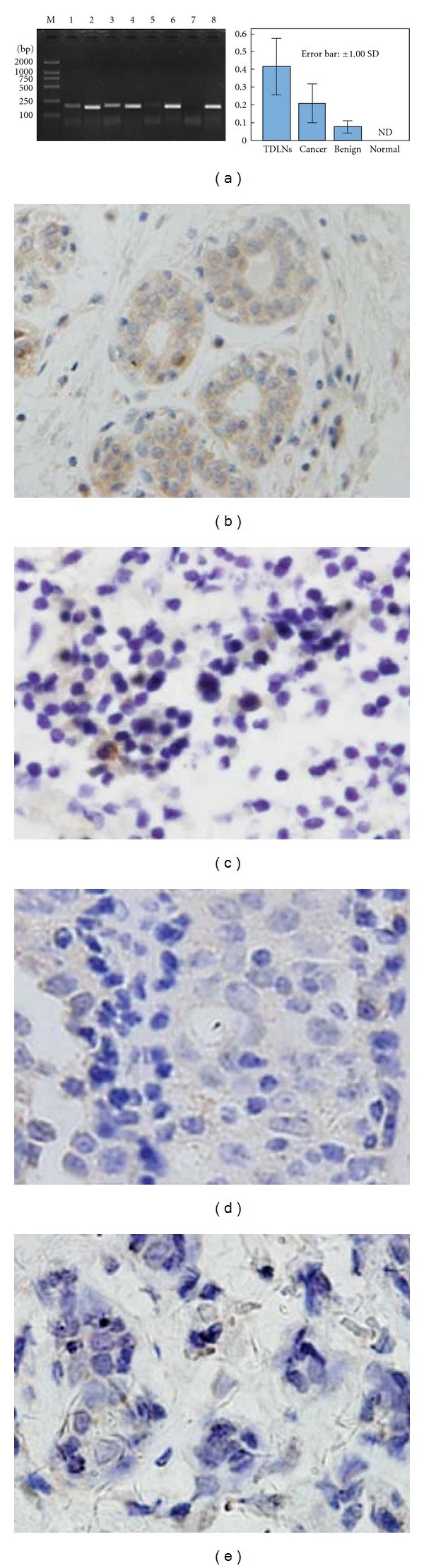
IDO expression in PTs and TDLNs was higher than that in benign diseases at both mRNA and protein levels. The expression of IDO in 26 cases of breast cancer PTs, TDLNs, and normal adjacent tissues and 10 cases of benign breast diseases was detected using qRT-PCR assay and IHC staining method. (a) The IDO mRNA expression in PTs was higher than that in benign diseases but lower than that in TDLNs using the grayscale density ratio of IDO/*β*-actin (lane 1: IDO in PTs; lane 2: *β*-actin in PTs; lane 3: IDO in TDLNs; lane 4: *β*-actin in TDLNs; lane 5: IDO in benign diseases; lane 6: *β*-actin in benign diseases; lane 7: IDO in normal tissues; lane 8: *β*-actin in normal tissues; M: DL2000 marker). (b)–(e) The IDO protein was predominantly expressed on cancer cells in breast cancer PTs (b) and in myeloid cell-like karyocytes in TDLNs (c), while less IDO expression was found in mammary ductal cells with hyperplasia in benign diseases (d). No IDO protein expression was detected in normal adjacent tissues (e).

The expression of IDO in 26 cases of breast cancer PTs, TDLNs, and normal adjacent tissues and 10 cases of benign breast diseases was detected using qRT-PCR and IHC methods. No detectable expression of IDO was observed in the normal adjacent tissues at either RNA or protein level (Figures 1(a) and 1(e)). The IDO mRNA expression in PTs was about 3 times higher than that in benign diseases by comparing the grayscale density ratio of IDO/*β*-actin (Figure 1(a), *P* < 0.05). Consistently, the IDO^+^SR and IDO^+^SI in PTs were significantly higher than that in benign diseases, which were 46.15% (12/26) versus 10.00% (1/10) for IDO^+^SR and 13.16 ± 7.82% versus 3.24 ± 1.30% for IDO^+^SI (Figures 1(b) and 1(c), *P* < 0.05). The mRNA expression of IDO in TDLNs was 2 times higher than that in PTs. Accordingly, the IDO^+^SR and IDO^+^SI in TDLNs were significantly higher than those in PTs which were 73.08% (19/26) versus 46.15% (12/26) for IDO^+^SR and 20.46 ± 6.57% versus 13.16 ± 7.82% for IDO^+^SI (Figures 1(b)–1(d), *P* < 0.05). Furthermore, we found that the IDO^+^ SIs in TDLNs were significantly positively correlated to those in PTs in a linear pattern as determined using the Regression Analysis (*r* ^2^ = 0.28, *P* < 0.05). Furthermore, all TDLNs collected from IDO^+^ primary tumors were positive for IDO staining when we used immunohistochemical cut-off value of 10% for IDO^+^ tumor cells. The mean IDO^+^SIs in positive tumors and corresponding TDLNs were 26.47 ± 14.12% and 33.97 ± 13.91%, respectively. Contrarily, the mean IDO^+^SIs in negative tumors and corresponding TDLNS were 5.56 ± 2.54% and 7.25 ± 3.43%, respectively. Therefore, comparing to the IDO^−^ tumors, the TDLNs collected from IDO^+^ tumors displayed higher level of IDO expression(*P* < 0.05) which had no correlation with the pathological type and multiple receptors(ER/PR/Her2) status of the primary tumors. However, higher IDO^+^SIs were observed in the metastatic TDLNs comparing to the nonmetastatic TDLNs, which were 34.41 ± 15.18% versus 21.45 ± 9.76% (*P* < 0.05). This result was consistent with the increase of IDO^+^ myeloid cell-like karyocytes and cancer cells in metastatic TDLNs.

### 3.2. The Expression of IDO in Breast Cancer PTs Was Positively Associated with the Clinical Staging and Lymph Node Metastasis of Tumors

In order to evaluate the clinical significance of IDO expression in breast cancer PTs, a univariate analysis was performed between the IDO^+^SI in PTs and corresponding clinical and pathological information of the same patient. As shown in Table 2, higher IDO^+^SI correlated with more advanced clinical staging and more extensive TDLNs metastasis. The IDO^+^SI in stage III breast cancer was significantly higher than those in stage II or stage I breast cancer, which were 22.47 ± 10.79%, 11.72 ± 6.48%, and 8.95 ± 3.79%, respectively (*P* < 0.05). Similarly, the IDO^+^SI in breast cancer with metastasis extended to N3 lymph nodes was significantly higher than those with metastasis limited to N2 and N1 lymph node or without lymph node metastasis, which were 28.35 ± 14.78%, 15.98 ± 7.14%, 11.42 ± 8.49%, and 10.29 ± 5.23%, respectively (*P* < 0.05). In contrast, there was no significant correlation between the IDO^+^ SI in PTs and other clinical and pathological indexes, such as age, menstrual status, tumor diameter, pathological type, histological grade, and expression of ER, PR, or Her2.

All patients were followed up at a median of 5 years, and the overall survival (OS) and time to progression (TTP) were analyzed. The mean OS of the IDO^+^ patients was shorter than that of the IDO^−^ patients (59.50 ± 5.01 m versus 86.15 ± 3.22 m), but the difference was not statistically significant (*P* value = 0.145). Similarly, the mean TTP of the IDO^+^ patients was shorter than that of the IDO^−^ patients (46.84 ± 3.29 m versus 78.91 ± 2.79 m), but the difference was not statistically significant (*P* = 0.147). Although it is difficult to demonstrate an inverse correlation between clinical prognosis and the IDO status, possibly due to the small sample size, our results implied a comparably worse outcome in IDO^+^ breast cancer patients.

### 3.3. The Expression of IDO in Breast Cancer PTs Was Positively Correlated with the Density of Tregs in PTs and TDLNs

**Figure 2:**
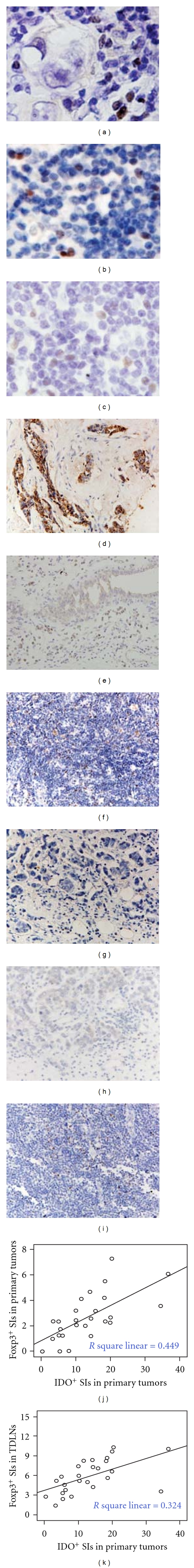
IDO expression in PTs was positively correlated with the density of Tregs in PTs and TDLNs. The Foxp3^+^ Tregs in PTs, TDLNs, benign disease, and normal adjacent tissues were detected using IHC staining method. (a)–(c) The Foxp3 protein appeared in the nuclei of lymphocytes infiltrated into PTs (a) and TDLNs, including nonmetastatic TDLNs (b) and metastatic TDLNs (c). (d)–(i) In the PTs with higher expression of IDO (d), more Foxp3^+^ Tregs infiltrated into the PTs (e) and corresponding TDLNs (f). In contrast, in the PTs with lower or no expression of IDO (g), less Foxp3^+^ Tregs were detected in the PTs (h) and corresponding TDLNs (i). (j) and (k) Scatter plots were generated to display the correlation between IDO expression in breast cancer (IDO^+^SIs in primary tumors) and the density of Foxp3^+^ Tregs in PTs (Foxp3^+^SIs in primary tumors) or in TDLNs (Foxp3^+^SIs in TDLNs). The IDO^+^SIs displayed a positive correlation with the Foxp3^+^ SIs in PTs with a linear regression equation of *Y* = 0.832 + 0.140 *X* (j). Accordingly, the IDO^+^SIs showed a similarly positive correlation with the Foxp3^+^ SIs in TDLNs with a linear regression equation of *Y* = 3.771 + 0.160 *X* (k).

The Foxp3^+^ Tregs in PTs, TDLNs, benign disease, and normal adjacent tissues were detected using IHC staining method. Foxp3 protein was detected in the nuclei of lymphocytes infiltrated into PTs and TDLNs, but seldom in benign breast diseases and in normal breast tissues (Figures 2(a)–2(c)). The Foxp3^+^ SIs in breast cancer PTs were significantly higher than those of benign breast diseases and normal breast tissues, which were 3.50 ± 1.04%, 0.71 ± 0.42%, and 0.55 ± 0.34%, respectively, (*P* < 0.05). In contrast, the Foxp3^+^ SIs in the PTs were significantly lower than those in the TDLNs which was 6.13 ± 2.31% (*P* < 0.05). More Foxp3^+^ Tregs infiltrated in the PTs with higher expression of IDO and corresponding TDLNs (Figures 2(d)–2(f)). Contrarily, in the breast cancer PTs with lower expression of IDO or absence of IDO expression, lower numbers of Foxp3^+^ Tregs were detected (Figures 2(g)–2(i)). The scatter plots were generated to display the correlation between IDO expression and density of Foxp3^+^ Tregs either in PTs or TDLNs. The results indicated that IDO expression in breast cancer was linearly correlated to the density of Treg in the PTs and TDLNs. Statistical analyses demonstrated that the IDO^+^ SIs displayed a positive correlation with the Foxp3^+^ SIs in PTs and TDLNs, with linear regression equations of *Y* = 0.832 + 0.140 *X* (*Y*: Foxp3^+^ SIs in PTs; *X*: IDO^+^SIs) (*r* ^2^ = 0.449, *P* < 0.05, Figure 2(j)) and *Y* = 3.771 + 0.160*X* (*Y*: Foxp3^+^ SIs in TDLNs; *X*: IDO^+^SIs) (*r* ^2^ = 0.324, *P* < 0.05, Figure 2(k)), respectively.

### 3.4. The Proportion and Absolute Number of CD4^+^CD25^+^CD127^−^ Tregs in CD3^+^ T Cells Increased After Coculturing with IDO/CHO Cells

**Figure 3:**
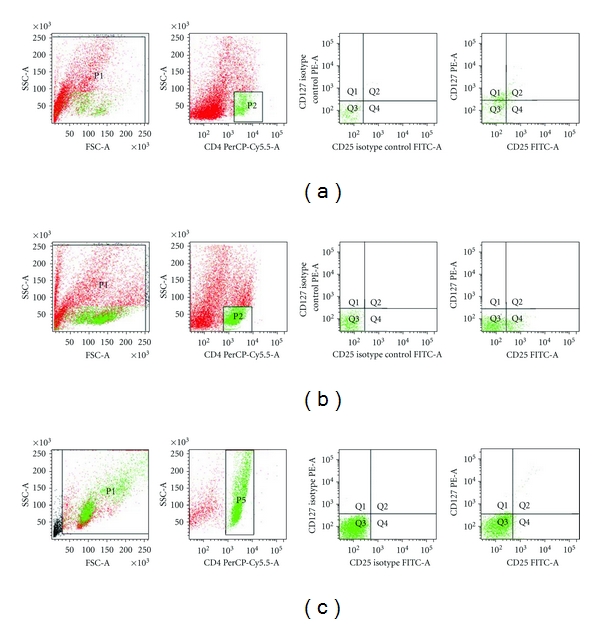
The proportion and absolute number of CD4^+^CD25^+^CD127^−^ Tregs in CD3^+^ T cells increased after coculture with IDO/CHO cells. CD3^+^ T cells isolated from PBMCs of breast cancer patients were cocultured with CHO/IDO or CHO/EGFP cells for 7 days to allow induction of Tregs. The proportions and absolute number of CD4^+^CD25^+^CD127^−^ Tregs were detected by flow cytometry. (a) The proportion of CD4^+^CD25^+^CD127^−^ Tregs in the T cells treated with IDO^−^ CHO/EGFP cells (P2 region represents CD4^+^ T cells; Q4 region represents CD4^+^CD25^+^CD127^−^ Tregs). (b) The proportion of CD4^+^CD25^+^CD127^−^ Tregs in the T cells treated with IDO^+^ CHO/IDO cells (P2 region represents CD4^+^ T cells; Q4 region represents CD4^+^CD25^+^CD127^−^ Tregs). (c) The CD3^+^ control T cells (P5 region represents CD4^+^ T cells; Q4 region represents CD4^+^CD25^+^CD127^−^ Tregs). The flow cytometry dot plots indicate data of one representative experiment. Each experiment was repeated at least 3 times.

The mRNA and protein expression of IDO, as well as the catalytic activity of tryptophan have been determined in CHO/IDO cells as reported in our previous study [23]. The CD3^+^ T cells isolated from PBMCs of breast cancer patients were cocultured with CHO/IDO and CHO/EGFP cells in complete medium supplemented with 10% FBS and 50 U/ml IL-2 for 7 days. The proportions and absolute number of CD4^+^CD25^+^CD127^−^ Tregs in treated and untreated T cells were detected using flow cytometry. The proportion of Tregs in CD4^+^ T cells increased from 3.43 ± 1.07% to 8.98 ± 1.58% after coculture with CHO/IDO cells, which is higher than that after coculture with CHO/EGFP cells (3.73 ± 1.12%) (*P* < 0.05, Figures 3(a)–3(c)). The absolute number of Tregs in CD3^+^ T cells stimulated by CHO/IDO was 629 ± 110.6 cells/*μ*L, higher than that in the coculture with CHO/EGFP cells (268 ± 80.6 cells/*μ*L) and that in unstimulated control CD3^+^ T cells (308 ± 96.3 cells/*μ*L) (*P* < 0.05).

### 3.5. The Expression of Foxp3 in CD3^+^ T Cells Was Upregulated at Both mRNA and Protein Levels After Coculturing with IDO/CHO Cells

**Figure 4:**
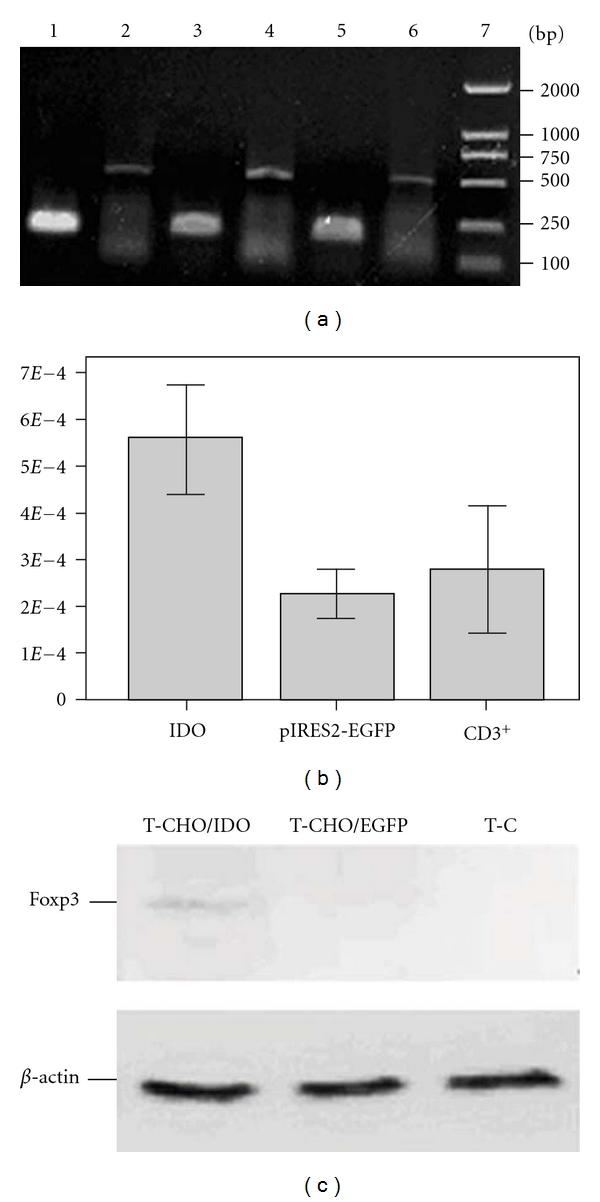
Foxp3 expression in CD3^+^ T cells was upregulated both at mRNA and protein levels after coculture with IDO/CHO cells. The expression of Foxp3 gene at mRNA and protein levels in treated and untreated T cells were detected using qRT-PCR assay and Western Blot method. (a) After 7 day coculture, the relative mRNA level of Foxp3 in the CD3^+^ T cells treated with the CHO/IDO cells was significantly higher than that in the CD3^+^ T cells treated with the CHO/EGFP control cells or untreated CD3^+^ T cells (lane 1: *β*-actin in the CD3^+^ T control cells; lane 2: Foxp3 in the CD3^+^ T control cells; lane 3: *β*-actin in the T cells treated with CHO/IDO cells; lane 4: Foxp3 in the T cells treated with CHO/IDO cells; lane 5: *β*-actin in the T cells treated with CHO/EGFP cells; lane 6: Foxp3 in the T cells treated with CHO/EGFP cells; lane 7: DL2000 Marker. (b) The mRNA amount of Foxp3 in the CD3^+^ T cells stimulated by the CHO/IDO cells was significantly higher than that in the CD3^+^ T cells stimulated by the CHO/EGFP cells and the unstimulated CD3^+^ T cells control. (c) Foxp3 expression at protein level in treated and untreated T cells detected by Western Blot analysis. Foxp3 expression was exclusively detected in the cell lysates of CD3^+^ T cells treated with CHO/IDO cells, indicating a 48 kD protein band reactive to a Foxp3-specific monoclonal antibody (lane 1: Foxp3 in CD3^+^ T cells treated with CHO/IDO cells; lane 2: Foxp3 in CD3^+^ T cells treated with CHO/EGFP cells; lane 3: Foxp3 in control CD3^+^ T cells).

The expression of Foxp3 gene at mRNA and protein levels in treated and untreated T cells were detected using qRT-PCR assay and Western Blot analysis. After 7 days of coculture, the relative mRNA amount of Foxp3 gene in the CD3^+^ T cells stimulated by the CHO/IDO cells was 0.00056 ± 0.00012, which was significantly higher than that in the CD3^+^ T cells stimulated by the CHO/EGFP cells (0.00023 ± 0.00005) and that in the unstimulated CD3^+^ T cells control (0.00028 ± 0.00013) (*P* < 0.05, Figures 4(a) and 4(b)). Furthermore, Foxp3 expression was exclusively detected in the lysates of CD3^+^ T cells stimulated by CHO/IDO cells, indicated by a 48 kD protein band reactive to a Foxp3-specific monoclonal antibody (Figure 4(c)).

## 4. Discussion

High level of IDO expression has been found in many malignant tumors, including colorectal cancer [24], endometrial cancer [25], lung cancer [26], ovarian cancer [27], and renal carcinoma [28]. However, its expression pattern in primary human breast cancer tissue has been seldomly reported. In this study we found that IDO expression at both mRNA and protein levels were significantly higher in breast cancer PTs and TDLNs than those in benign diseases. IDO was mainly expressed in cancer cells in breast cancer PTs and expressed at lower levels in certain myeloid cell-like karyocytes in TDLNs and hyperplastic ductal cells in benign diseases, but not expressed in normal adjacent tissues. Our results demonstrated that the IDO^+^SIs in breast cancer PTs were significantly higher in tumors of more advanced stages and with more extensive lymph node metastasis, which correlated with a comparably worse clinical outcome. These results suggest that IDO may play a pivotal role in promoting metastasis of breast cancer, as the IDO-positive breast cancer cells seem to have a higher potential in migrating to axillary lymph nodes than the IDO-negative ones.

The above results coincided with the previous reports that more extensive IDO expression in primary cancer tissues was associated with higher distant metastasis rate in clinic [24, 25]. A study by Sakurai et al. indicated that high expression of IDO in breast cancer correlated with clinical stage and may therefore play a critical role in immunosuppression in those patients [29]. However, the mechanisms involved in this pathogenesis process remain unknown. It is proposed that local T-cell-based immunotolerance induced by high level of IDO in the tumor microenvironment might be the predominant immunoregulatory mechanism facilitating tumor metastasis [24]. As a tryptophan catabolic enzyme, IDO and metabolites have been reported as key regulators in suppressing immune surveillance and inducing immunotolerance in several diseases [30]. Several mechanisms by which IDO contributes to immune escape have been identified. IDO suppresses proliferation of T cells by hampering cell cycle in mid-G1 phase [31]. IDO also promotes apoptosis of activated T cells which were more sensitive to Fas-dependent apoptosis after tryptophan deprivation [32]. Furthermore, IDO has been reported to inhibit T-cell-mediated immune response by directly inducing the differentiation of CD4^+^CD25^−^ T cells into CD4^+^CD25^+^ Tregs, or directly activating mature Tregs [33]. 

CD4^+^CD25^+^ Tregs are a subset of regulatory T cells with potent inhibitory effects on innate and adaptive immunity both in physiological and pathological status which play important roles in tumor evasion and metastasis [34, 35]. It is currently accepted that Foxp3 is the most specific marker in Tregs which plays crucial roles in the generation and function of Treg [36]. In our study, the Foxp3^+^ SIs in breast cancer PTs were significantly higher than those in benign diseases and normal adjacent tissues, but lower than those in the TDLNs which showed the same pattern as the IDO^+^SIs. Therefore, we studied the correlation between the expression levels of IDO and the density of Tregs both in breast cancer PTs and TDLNs. Our data demonstrated that in the IDO-positive breast cancer samples, more Tregs infiltrated into the PTs and TDLNs, compared to the IDO-negative ones. In addition, the expression of IDO in breast cancer PTs was positively linearly correlated to the density of Treg in the PTs and TDLNs in linear regression analysis. To find out if high level of IDO could induce amplification of Foxp3^+^Tregs, we cocultured CD3^+^ T cells with IDO^+^ CHO(IDO/CHO) cells* in vitro*. We found that the proportion and absolute number of CD4^+^CD25^+^CD127^−^ Tregs increased after coculturing CD3^+^ T cells with IDO/CHO for 7 days in which Foxp3 expression was upregulated at both mRNA and protein levels. These results implied that upregulated IDO in CHO cells might favor amplification of CD4^+^CD25^+^CD127 Tregs and induce increasing expression of Foxp3 both *in vivo* and *vitro* which coincided with the previous report that the long-term effect of the catabolic products of tryptophan is to enable the regulatory function of CD4^+^CD25^−^ T cells by inducing Foxp3 expression and secreting inhibitory cytokine TGF-*β* [37]. 

It is has been indicated that the interaction between IDO and Tregs is a mutual effect, in which high level of IDO promotes the differentiation, activation, and maturation of Tregs; conversely, the CTLA4 constitutively expressed on CD4^+^CD25^+^ Tregs significantly stimulates synthesis and increases enzyme activity of IDO by binding to CD80/CD86 on dendritic cells (DCs) [38]. This theory was supported by the observations from Munn's group which indicate that overexpression of IDO in antigen-presenting cells (APCs) was the major cause of tumor-derived immune tolerance in local lymph nodes of patients with breast cancer or melanoma [39]. Consistently, in this study we also observed that some IDO high-expressing myeloid cell-like karyocytes in TDLNs displayed positively linear correlation to the IDO expression on cancer cells in PTs, which might participate in the mutual cross-talk between IDO^+^ cancer cells and Foxp3^+^ Tregs and further magnified the immunosuppressive cascade triggered by IDO. 

However, Jacquemier et al. reported an opposite effect of IDO in medullary breast cancer (MBC), a subtype of basal-like breast cancer different from invasive carcinoma, in which high expression of IDO in stromal or epithelial cells was associated with large amount of lympoid infiltrate and a favorable clinical outcome of patients [40]. This report, however, stated that the beneficial prognosis of IDO^+^ tumors was exclusively observed in basal-like breast cancer, but not in other subtypes of breast cancer. Therefore, the discrepancy between this previous study and ours may be attributed to the different pathological subtypes of breast cancer examined. In our study, most samples were invasive ductal or lobular carcinomas, and no basal-like breast cancers are included. Our conclusion is consistent with the study of Mansfield et al. using 47 cases of breast cancer samples, including 25 invasive ductal carcinoma and 18 invasive lobular carcinoma, where IDO^+^ sentinel lymph nodes accompanied by infiltration of Foxp3^+^ Tregs imply lymph node metastasis of breast cancer, and are therefore regarded as a negative prognostic factor [12]. 

In conclusion, our study implied that upregulation of IDO in breast cancer cells might inhibit local immune surveillance by favoring amplification and infiltration of CD4^+^CD25^+^ Tregs in the tumor microenvironment and thus promote metastasis and relate to a bad prognosis. Therefore, novel and efficient IDO-targeted therapies may provide a new strategy of breast cancer treatment.

## Figures and Tables

**Table 1:** Primers for real-time quantitative RT-PCR.

Gene name	Primer sequences	Product size
IDO	UP: 5′-CATCTGCAAATCGTGACTAAG-3′	188 bp
	DP: 5′-CAGTCGACACATTAACCTTCCTTC-3′	

Foxp3	UP: 5′-CCCACTTACAGGCACTCCTC-3′	486 bp
	DP: 5′-CTTCTCCTTCTCCAGCACCA-3′	

*β*-actin	UP: 5′-TGGCACCCAGCACAATGAA-3′	186 bp
	DP: 5′-CTAAGTCATAGTCCGC CTAGAAGCA-3′	

Note: UP: upstream primer; DP: downstream primer.

**Table 2:** The relationship between IDO expression and clinical pathological indexes.

	*N*	IDO^+^SI (%)	*P* value
Age (years)			
<60	18	12.30 ± 8.35	0.465
≥60	8	15.11 ± 10.75	

Menstrual status			
Postmenopausal	14	15.02 ± 10.93	0.257
Nonpostmenopausal	12	10.83 ± 5.42	

Tumor diameter (cm)			
≤2	7	11.89 ± 6.55	0.211
2*∼*5	15	13.43 ± 6.88	
>5	4	14.38 ± 7.69	

Clinical stage			
I	2	8.95 ± 3.79	0.034
II	20	11.72 ± 6.48	
III	4	22.47 ± 10.79*	

Pathological type			
Invasive Ductal Ca.	21	13.62 ± 9.39	0.223
Others	5	11.23 ± 6.25	

histological grade			
I	8	10.73 ± 6.45	0.324
II	10	12.29 ± 7.28	
III	8	13.71 ± 5.96	

TDLNs metastasis			
pN0	6	10.29 ± 5.23	0.046
pN1	14	11.42 ± 8.49	
pN2	4	15.98 ± 7.14	
pN3	2	28.35 ± 14.78*	

ER status			
(−)	12	15.76 ± 10.58	0.394
(+)	14	12.70 ± 8.83	

PR status			
(−)	13	13.18 ± 8.02	0.624
(+)	13	15.29 ± 9.31	

Her-2 status			
(−)	18	11.69 ± 8.46	0.457
(+)	8	14.34 ± 10.23	

*Statistically significant difference between the samples of advanced stage (stage III) and earlier stage (stage II and stage I), as well as the significant difference between the samples with more extensive LN metastasis (pN3) and less or no LN metastasis (pN0-2).
